# A163 CATCHING CANCERS EARLY? AGE AND STAGE AT DIAGNOSIS OF COLORECTAL CANCER IN FIRST NATIONS PEOPLE IN ONTARIO COMARED TO OTHER ONTARIANS

**DOI:** 10.1093/jcag/gwad061.163

**Published:** 2024-02-14

**Authors:** J Tinmouth, R Sutradhar, N Liu, L Senese, S Leylachian, A Chiarelli, A Kewayosh, R Kupets, M Tammemagi, A Sheppard

**Affiliations:** Sunnybrook Health Sciences Centre, Toronto, ON, Canada; Institute for Clinical Evaluative Sciences, Toronto, ON, Canada; Institute for Clinical Evaluative Sciences, Toronto, ON, Canada; Sunnybrook Health Sciences Centre, Toronto, ON, Canada; Ontario Health, Toronto, ON, Canada; Ontario Health, Toronto, ON, Canada; Ontario Health, Toronto, ON, Canada; Ontario Health, Toronto, ON, Canada; Ontario Health, Toronto, ON, Canada; Ontario Health, Toronto, ON, Canada

## Abstract

**Background:**

Historically, Indigenous peoples in Canada were at lower risk for cancer compared to non-Indigenous people. Data from prior to 2010 suggested that First Nations in Ontario had higher incidence & poorer survival from colorectal cancer (CRC). The current project arose from concerns raised by Indigenous partners about community members being diagnosed with colorectal cancer before screening age-eligibility.

**Aims:**

To examine the age & stage at CRC diagnosis among First Nations people in Ontario (FN) compared to other Ontarians (ON).

**Methods:**

With Indigenous partner support, approvals were obtained to link health administrative datasets at ICES & Ontario Health. Among others, these datasets included the Ontario Cancer Registry & the Indian Register System, a registry of FN with status, meaning they are on the official record of persons registered under section 6 of Canada’s Indian Act. We created cohorts of FN & ON aged 18-85 from 2000 to 2018, matched 1:5 on sex, age at & year of inception. For the primary analysis, we performed survival analysis, accounting for the competing event of death & using attained age as a time scale. The primary outcome was CRC diagnosis, the primary exposure was FN vs ON & we adjusted for regular screening test use, comorbidity, remoteness & having a regular primary care provider. Similarly, we examined stage at diagnosis, restricting the cohort to 2007+ due to limitations in the stage data.

**Results:**

Baseline demographics for the 153,300 FN & 766,500 ON included are in the Table. Median (IQR) follow-up was 17 (8-20) & 18 (I8-20) years for FN & ON, respectively (p ampersand:003C0.001). There were 1,156 (0.8%) & 4,249 (0.6%) CRCs diagnosed in FN & ON, respectively (p ampersand:003C0.001). 11,213 (7.3%) FN & 25,732 (3.4%) ON died from other causes (p ampersand:003C0.001). After accounting for the competing event of death, FN were significantly more likely to be diagnosed with CRC at a younger age (HR 1.42, 95%CI: 1.32-1.53) (see Figure) & later stage (HR 1.53, 95%CI: 1.34-1.75) than ON.

**Conclusions:**

FN with CRC were younger & presented at a later stage compared to ON with CRC. These findings have important implications for CRC screening recommendations for First Nations people in Ontario.

Baseline characteristics at cohorts inception

IQR, interquartile range

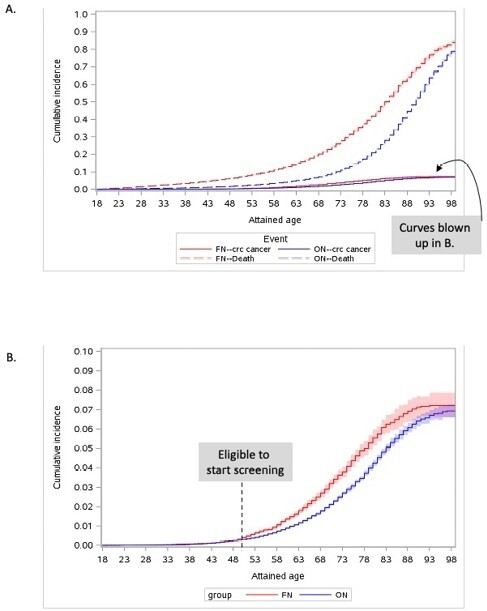

A. Cumulative incidence of colorectal cancer (primary event) & death (competing event), comparing First Nations (FN) and other Ontarians (ON). B. Focus on cumulative incidence of colorectal cancer (death as competing event, not shown), comparing FN and ON.

**Funding Agencies:**

CIHR

**Table:** 

	First Nations People n=153,300	Other Ontarians n=766,500
Median age (IQR)	26 (18-40)	26 (18-40)
Male Sex, No. (%)	78,078 (51%)	390,390 (51%)
Regular screening program test use, No. (%)	991 (0.6%)	9,285 (1.2%)
Major comorbidities, No. (%)		
0	37,544 (25%)	532,792 (80%)
1	37,544 (25%)	178,892 (23%)
2+	17,721 (12%)	54,816 (7%)
Rural, No. (%)	71,875 (47%)	87,571 (11%)
No regular PCP, No. (%)	44,794 (29%)	129,337 (17%)

